# Lyotropic Liquid Crystal (LLC)-Templated Nanofiltration Membranes by Precisely Administering LLC/Substrate Interfacial Structure

**DOI:** 10.3390/membranes13060549

**Published:** 2023-05-24

**Authors:** Senlin Gu, Liangliang Zhang, Liliana de Campo, Luke A. O’Dell, Dong Wang, Guang Wang, Lingxue Kong

**Affiliations:** 1 Institute for Frontier Materials, Deakin University, Geelong, VIC 3216, Australia; gusen@deakin.edu.au (S.G.); zhangliang@deakin.edu.au (L.Z.); luke.odell@deakin.edu.au (L.A.O.); 2 Australian Centre for Neutron Scattering, Australia Nuclear Science and Technology Organization (ANSTO), Sydney, NSW 2234, Australia; lilianad@ansto.gov.au; 3 Hubei Key Laboratory of Advanced Textile Materials & Application, Hubei International Scientific and Technological Cooperation Base of Intelligent Textile Materials & Application, Wuhan Textile University, Wuhan 430200, China; Wangdon08@126.com; 4 Institute of High Energy Physics, Chinese Academy of Sciences, Beijing 100049, China; 5 Spallation Neutron Source Science Centre, Dongguan 523803, China

**Keywords:** lyotropic liquid crystal, nanofiltration, interfacial structure, surface modification

## Abstract

Mesoporous materials based on lyotropic liquid crystal templates with precisely defined and flexible nanostructures offer an alluring solution to the age-old challenge of water scarcity. In contrast, polyamide (PA)-based thin-film composite (TFC) membranes have long been hailed as the state of the art in desalination. They grapple with a common trade-off between permeability and selectivity. However, the tides are turning as these novel materials, with pore sizes ranging from 0.2 to 5 nm, take center stage as highly coveted active layers in TFC membranes. With the ability to regulate water transport and influence the formation of the active layer, the middle porous substrate of TFC membranes becomes an essential player in unlocking their true potential. This review delves deep into the recent advancements in fabricating active layers using lyotropic liquid crystal templates on porous substrates. It meticulously analyzes the retention of the liquid crystal phase structure, explores the membrane fabrication processes, and evaluates the water filtration performance. Additionally, it presents an exhaustive comparison between the effects of substrates on both polyamide and lyotropic liquid crystal template top layer-based TFC membranes, covering crucial aspects such as surface pore structures, hydrophilicity, and heterogeneity. To push the boundaries even further, the review explores a diverse array of promising strategies for surface modification and interlayer introduction, all aimed at achieving an ideal substrate surface design. Moreover, it delves into the realm of cutting-edge techniques for detecting and unraveling the intricate interfacial structures between the lyotropic liquid crystal and the substrate. This review is a passport to unravel the enigmatic world of lyotropic liquid crystal-templated TFC membranes and their transformative role in global water challenges.

## 1. Introduction

The reduction in the supply of freshwater has prompted governments and scientists to develop water purification technologies [1]. As seawater makes up 96.5% of the total global water supply, seawater desalination is the most promising way to increase potable water supplies [2]. However, desalination consumes 6–7 times more energy than conventional water treatment methods to purify an equivalent amount of water [3]. In a common desalination plant, the membrane unit accounts for 85% of the overall energy consumption [4]. Therefore, improving membrane efficiency is crucial in reducing overall desalination energy consumption and further popularizing desalination techniques.

Thin-film composite (TFC) membranes, consisting of an active separation layer, porous support, and polyester non-woven backing layer, are considered a state-of-the-art design for seawater desalination membranes [5,6,7]. The active layer of commercial TFC membranes is typically a nonporous aromatic cross-linked polyamide (PA) [8]. Although this type of TFC is widely used, it is limited by its low permeability (<1 Lm^−2^ h^−1^ bar^−1^ μm) [9,10], non-uniform pore size [11], and low fouling resistance [7]. Lyotropic liquid crystals (LLCs) are promising templates for active layer materials due to their inherently uniform and controllable pore size, ranging from 0.2 to 5 nm [9,12,13,14,15,16]. Membranes formed by LLC template materials possess low surface roughness and high hydrophilicity, which result in higher membrane-fouling resistance [17]. Moreover, mesophases such as hexagonal and lamellar are reorientable, enabling water channels to align perpendicularly to the membrane surface and increasing water permeance.

Current studies on fabricating TFC membranes based on the LLC template active layer all directly form the LLC mesophase on substrates since this method guarantees interface [10]. However, research has shown that the surface properties of the substrate, including hydrophilicity and heterogeneity, could significantly affect the types and shapes of surfactant mesophases [18]. As LLCs are also formed by surfactants, it is reasonable to wonder about the accurate structures at the LLC/substrate interface. However, this essential part has been overlooked.

Studies on the conventional PA-based active layer show that the middle porous substrate of TFC not only regulates the water transport path but also interferes with the structure formation of the active layer [19]. For example, the funnel effect leads to a longer transport pathway of water on the substrate with low porosity [20,21]. Regarding structure formation interferences, the nanovoids formation on the PA surface has been effectively controlled by the surface pore size of the substrates, which can be explained by a volcano-like model [22] and the confinement effect [23,24]. In addition, the infusion depth of the PA layer in the substrate was found to be altered by both surface pore size and hydrophilicity [25,26,27]. Commercial polymer substrates are usually limited by their material nature, which is hydrophobic, has less porosity, and cannot enable the top layer to exert functions effectively.

Fortunately, the limitations of polymer substrates can be remedied by emerging surface modification and by introducing an interlayer between the substrate and top layer [28,29]. Surface modification methods, including surface deposition and surface grafting, usually focus on tackling the polymer surface hydrophobicity [29], while introducing an interlayer could bring additional benefits, such as adjusting the overall water channel [30], restricting the active layer infusion, and facilitating the formation of a uniform and defect-free top layer [31].

In addition to optimizing the surface properties of the substrates, accurately detecting the LLC/substrate interfacial structures is also of great importance for optimizing the interface. Microscopic methods such as scanning electron microscopy and transmission electron microscopy are usually destructive to the membranes and limited to probing small sample areas. X-ray and neutron techniques are more promising in non-destructively observing the buried LLC/substrate interfacial structures. The grazing incidence mode scattering technique is the most direct tool to identify the LLC phase structure at the interface by choosing a suitable beam incident angle [32], while neutron enables localization of the monomer molecules at the interface by contrast matching the deuterated amphiphile molecules and the mixtures of deuterated and hydrogen solvents [33].

While the use of LLC mesophases in advanced materials has been observed in various fields, the effects of the bottom substrate on the formation and dimensions of the LLC phase structure have not been systematically discussed. Moreover, there is a lack of discussion on the design of the LLC/substrate interface and the accurate detection of this interface. To address this gap in knowledge, this review aims to critically examine and evaluate the effects of substrates on both active layer formation and the TFC water path, and to systematically summarize the related mechanisms. Specifically, we compare the conventional PA active layer and LLC active layer and provide an optimized substrate recommendation for both cases. We also suggest the corresponding substrate modification methods and interfacial characterization tools to support the administration of the interfacial layer.

## 2. Fabrication of LLC Active Layer with Suitable Structure Retention

The structure of TFC membranes based on the LLC template active layer typically consists of a top LLC template layer and a porous substrate. The porous substrates are typically commercially purchased microfiltration or ultrafiltration membranes or self-prepared (phase inverse method) porous membranes. This section presents the main efforts made in the fabrication of the LLC active layer on porous membranes, focusing on LLC phase behavior, structure retention, and a comparison of the resulting membranes structures and performances.

### 2.1. LLC Precursor on Porous Substrates

#### 2.1.1. LLC Phases

**Figure 1:**
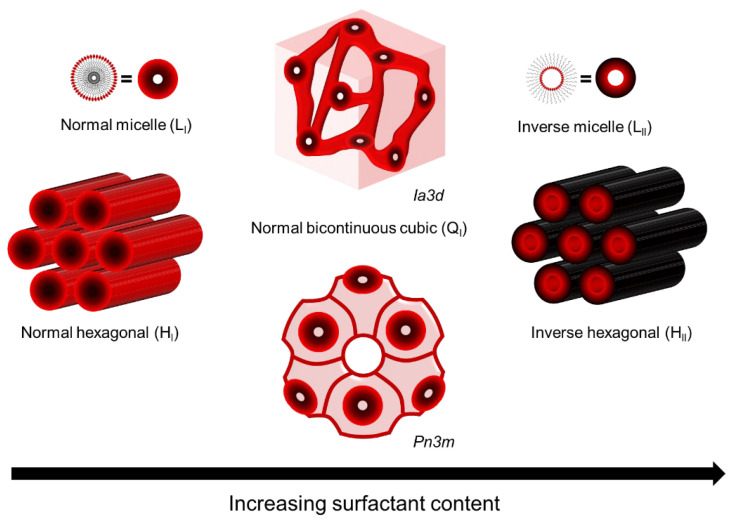
LLC phases used as template for fabricating top layer.

LLCs are assemblies composed of amphiphilic molecules that contain a hydrophilic headgroup and a hydrophobic organic tail in the presence of a polar solvent, usually water. The amphiphilic nature of these molecules drives them to phase separation upon the addition of polar solvent, resulting in the formation of ordered hydrophobic and hydrophilic domains with water–oil interfaces determined by hydrophilic headgroups. The most common phases of LLC are lamellar (L), hexagonal (H), bi-continuous cubic (Q), and discontinuous cubic (I). Among these, the normal hexagonal phase (H_I_), inverted hexagonal phase (H_II_), and normal bi-continuous cubic (Q_I_) are most commonly used as the template for fabricating top layers [10,16,34,35], as shown in Figure 1. Saadat and his coworkers have summarized the reactive and non-reactive surfactants that have been used to form these mesophases [17]. Compared to other mesophases, the hexagonal mesophase template materials are more promising for fabricating top layers with pores parallel to the macroscopic transport direction, i.e., vertical to the membrane surface. Such a tubular HLLC domain was verified to be reoriented using several up-to-date techniques, including magnetic field [14,36,37,38,39], electrical fields [40,41,42], shear force [10,12,43,44,45,46], and confinement methods [13,47].

#### 2.1.2. LLC Phase Behavior

A lipid monolayer possessing a spontaneous curvature can be bent with a concave surface to form cylinders, which is the basis of the LLC phase transition. The free energy of bending a thin surfactant layer can be calculated by [48]:

(1) $$E={\left(k_{c}/2)(C_{1}+C_{2}-C_{0}\right)}^{2}+k_{g}C_{1}C_{2}$$

where *E* is the energy per lipid area, *C*_1_ and *C*_2_ are the principal curvatures, *k_c_* and *k_g_* are constants called the rigidity and Gaussian curvature constants, and *C*_0_ is referred to as the spontaneous curvature. *C*_1_, *C*_2,_ and *C*_0_ are zero for the lamellar phase, and *C*_1_ + *C*_2_ = 0 for the bi-continuous cubie phase, while in the hexagonal phase, one of the principle curvatures is zero [2,48]. Therefore, for the cylinder phase, the free energy of bending a thin surfactant layer can be transferred to [48]:

(2) $$E=(k_{c}/2){\left(C-C_{0}\right)}^{2}$$

where lower free energy of bending a thin layer corresponds to a higher spontaneous *C*_0_. However, this theory cannot explain the energy changes during the phase transition between the lamellar and hexagonal phases since the *C*_0_ is zero for the lamellar phase. In this case, the hydrocarbon packing energy theory was introduced [49,50,51]. The lamellar phase possesses a low hydrocarbon packing energy and a high bending energy, which means that the lower hydrocarbon packing energy drives the phase transition from the lamellar to the hexagonal phase when increasing the *C*_0_ of the flat layer. Therefore, the competition between the curvature on the interface of the mesophases and hydrocarbon packing mainly determines the structure of the LLC phase [48]. The temperature [52], pressure [53,54], pH value [55], and introducing agents such as inorganic salts [56,57] and alkanes [58] have been found to modulate the packing and bending energies of the LLC system. For instance, the decrease in temperature was found to increase the radius of the water core of the H_II_, which increases the hydrocarbon packing energy and forms a cylinder with a lower spontaneous curvature of the system. In this case, the introduction of a long-chain alkane can decrease the packing energy and increase the spontaneous curvature of the system, which stabilizes the LLC phase [48]. Efforts to control the LLC phase structure, LLC unit cell size, and diameter of the water phase in LLC have been motivated by many applications, including drug release and protein loading [59,60,61].

#### 2.1.3. Polar Solvent

Water is the most typical polar solvent used in LLC mesophase. However, the inevitable evaporation of water limits the retention of LLC phase structures. This problem can be addressed by replacing the water with other polar organic solvents with lower volatility, such as glycerol, ionic liquids, ethylene glycol, N-methylsydone, formamide and its derivatives, and propylene carbonate [62,63,64]. These solvents have been studied to form LLC phases with alternative surfactants.

### 2.2. Ultrathin LLC Film Formation on Porous Substrates

#### 2.2.1. LLC Templating

LLC mesophases suffer from poor mechanical and thermal properties, which limit their applications. Therefore, they are often used as templates to be polymerized into polyLLCs to overcome these limitations. Two common routes can be implemented to achieve robust polyLLCs: synergistic and transcriptive templating. The synergistic method is more direct since it cures the polymerizable surfactant directly, while the latter one cures the introduced monomers or crosslinkers in the confined area in the LLC template. Generally, self-assembly is a process by which molecules move to minimize their free energy. When transferring the LLC parent template to the polymer, the entropy of the system decreases, while the enthalpy of the isolated system commonly undergoes almost no change during the polymerization. Therefore, an increase in the free energy of the system will inevitably occur, leading to the collapse of the template structure. Two strategies can be used to avoid the loss of the parent structure [65]:

- Establishing strong enough thermodynamic interactions between the surfactant template and polymer;
- Increasing the system viscosity and chain entanglement by forming covalent and limiting species diffusion.

The fast formation of covalent bonds between monomers and the increase in the viscosity of the system by polymerization are reported to be the best strategies to retain the LLC structure, which restricts the diffusivity of the objects in the system constantly. Compared to synergistic templating, retaining the structure during the curing is more challenging for transcriptive templating since there are more movable objects in the transcriptive systems. Even so, a suitable choice of chemistry structure for monomers and initiators and the introduction of crosslinkers, polymerizable surfactant [66], silica network [67], and block copolymer [68] can increase the possibility of structure retention.

#### 2.2.2. Fabrication Process

**Figure 2:**
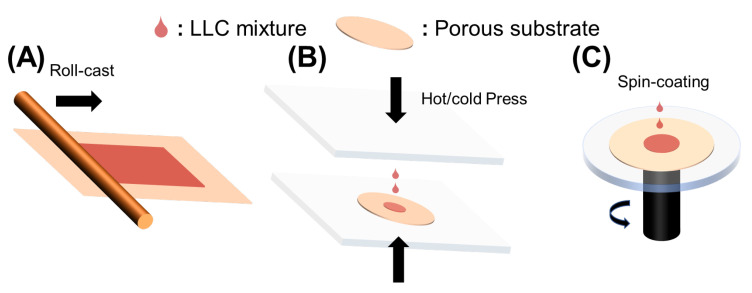
Methods to fabricate LLC-based TFC membranes. (**A**) Roll-casting; (**B**) hot or cold pressing; (**C**) Spin-coating.

Roll-casting, hot or cold pressing, and spin-coating were used to fabricate LLC-based TFC membranes, as shown in Figure 2A. To fabricate a robust LLC-based TFC membrane, the inverse hexagonal (H_II_) phase was first roll-cast onto the commercial microporous polysulfone (Psf) support. Although this fabrication method is facile, the inevitable evaporation of water causes limited retention of H_II_ phase structures. The use of alternative solvents has been discussed in Section 2.1.3.

The pressing method was developed to prevent solvent evaporation, as shown in Figure 2B. This method effectively reduces the possibility of water evaporation affecting TFC performances, but it leads to the infusion of the active layer into the porous support, making the active layer as thick as the support. This problem can be resolved by reducing the pore size of the porous support, improving the hydrophilicity of the substrate, decreasing the pressing temperature (need to ensure the phase formation in the specific temperature), and introducing a sacrificial layer. The sacrificial layer method has been developed to fabricate thin free-standing films and avoid infusion of LLC mesophase into the substrate. Generally, water-soluble polymers, such as polyvinyl alcohol (PVA), poly (vinylpyrrolidone) (PVP), poly (4-vinylphenol), and dextran, act as the sacrificial layers [10,12,69,70]. The main drawback of this method is water solvent absorption from the water-soluble polymer during the fabrication process, which can greatly influence the LLC phase structures. Thus, this method is only suitable for LLC systems with selective polar organic solvents.

Spin-coating (Figure 2C) is a straightforward method for producing ultrathin and uniform films, with membrane thickness controlled by solution concentration and spin speed [71]. However, for the fabrication of LLC membranes, it is crucial to maintain control over the LLC phase structure during the high spin speeds [10]. The structures and alignment [72] can be significantly altered by changes in spin speed, solvent, and even airflow direction for solvent evaporation. Moreover, a low-volatility polar organic solvent is necessary to form the LLC phase using this method.

Economic costs and life-cycle emissions are important in chemical use, fabrication, and operation needs for application [73]. Herein, economic costs and life-cycle emissions during the membrane fabrication process play important roles in future applications. From an economic cost perspective, interfacial polymerization can be a more complex and specialized process. It often requires specific equipment, chemicals, and controlled reaction conditions, which can contribute to higher upfront costs and investment in infrastructure. The cost of raw materials and the complexity of the production process can affect the overall economic feasibility of interfacial polymerization-based applications. Compared to the interfacial polymerization of PA-based TFC, the fabrication of LLC-based TFC can be more cost-effective. The three methods discussed above require less specialized equipment. Chemicals used in LLC systems are of fewer types and possess higher stability than that used in PA. Additionally, solid-like LLC precursor usually forms before membrane fabrication. This pre-polymerization process determines better reaction conditions controlled by free-radical polymerization than interfacial polymerization. From a life-cycle emission perspective, both interfacial polymerization and free-radical polymerization process produce emissions, including volatile organic compounds, greenhouse gases, and other chemical pollutants. These emissions can be mitigated by various strategies, such as optimizing reaction conditions and introducing efficient catalysts or initiators.

### 2.3. Performance of TFC Membranes with LLC as the Active Layer

Many LLC template membranes currently outperform the previous ones limited by trade-off, and their performances are comparable to commercial membranes (Table 1). Studies have mainly focused on building the outperformed TFCs with LLC active layers using hexagonal columnar LLC and cubic LLC. Gin and his coworkers reported a TFC with an inverted hexagonal (H_II_) phase as the active layer and polysulfone (Psf) UF as the substrate [34]. This H_II_ active layer with 0.6 μm possesses a molecular separation pore size of about 1.2 nm. However, the isotropic cylindrical nanochannels without alignment presented a very low pure water flux of 0.053 Lm^−2^ h^−1^ bar^−1^ μm. Osuji et al. [10] reported a TFC membrane with a parallel aligned hexagonal (H_I_) phase active layer. In this study, a spin-coated ultrathin (200 nm) active layer provided a relatively high water flux of 2 Lm^−2^ h^−1^ bar^−1^ μm. The molecular weight cutoff (MWCO) of this TFC membrane is 300 Da for charged organic solutes and 600 Da for neutral organic solutes (1.2 nm PEG). The salt (MgCl_2_ and CaCl_2_) rejection of this membranes is more than 80%.

The standard for salt rejection for drinkable desalinate seawater is more than 95% [77], and the hydrated Na^+^ is about 0.72 nm [5,78]. However, it is very challenging to fabricate a hexagonal mesophase with continuous and uniform nanopores in a diameter range smaller than 1 nm. Gin and his coworkers reported TFC membranes based on the bi-continuous cubic (Q_I_) phase [16,35]. The effective pore size of the active layers was found to be 0.75–0.96 nm, which allows for a 95% NaCl rejection. By replacing the water solvent with organic solvent glycerol, NaCl rejection was improved to 98% by significantly decreasing the defects in the active layer. However, the low effective pore size (<1 nm) of the Q_I_ mesophase leads to low water flux (<0.1 Lm^−2^ h^−1^ bar^−1^ μm) [63].

## 3. Effect of Substrate on TFC’s Structures and Performance

The porous substrate plays an important role in not only mechanically supporting the active layer but also exerting great effects on its structural control and filtering performance. Porous substrates are usually fabricated from polymer materials with various properties [79,80]. Polymers such as polysulfone (Psf) and polyether sulfone (PES) are most widely used as substrate materials in TFC membranes. They possess relatively high hydrophilicity and suitable thermal properties, but their relatively low mechanical strength and solvent sensitivity limit their application. Polyacrylonitrile (PAN) possesses higher hydrophilicity compared to other materials, but its relatively low mechanical strength is a limitation. Materials such as polyvinylidene fluoride (PVDF) and polypropylene (PP) possess high resistance to various chemicals, but they suffer from high hydrophobic surfaces. Polyvinyl chloride (PVC) possesses high mechanical strength but poor thermal stability [46,81]. The performance of TFC was reported to be greatly influenced by the surface pore structures, hydrophilicity, and roughness of the support.

### 3.1. Substrate’s Effect on Traditional TFC’s Structures and Performances

#### 3.1.1. Pore Structures

**Figure 3:**
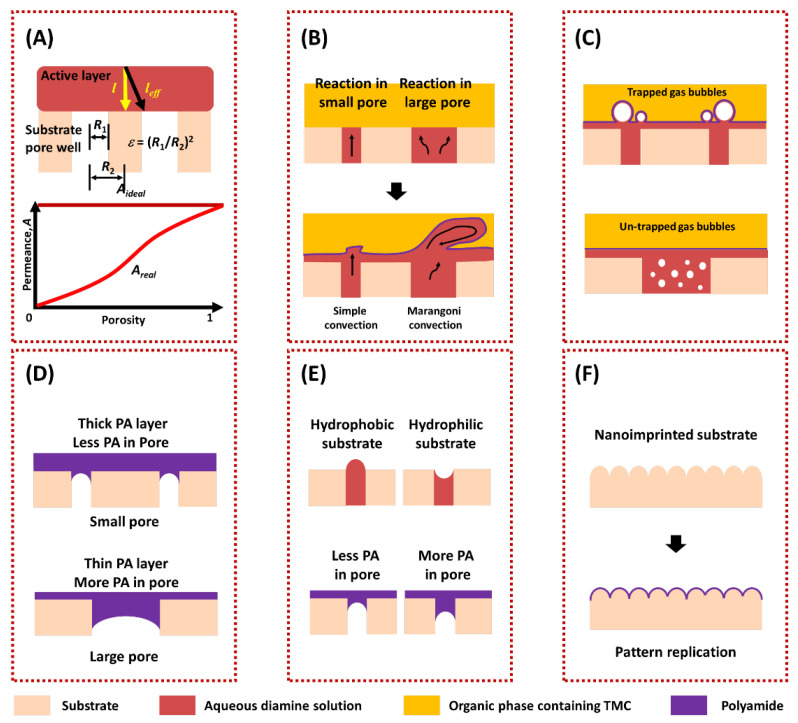
(**A**) The relationship between the permeance of the TFC membranes and the porosity of the substrates. (*l*: intrinsic thickness of the active layer, *l*_eff_: effective transport length, *ε*: porosity, *R*_1_: mean pore radius, and *R*_2_: mean half-distance between pores) [19]; (**B**) the relationship between the pore size of the substrate and roughness of the resulting PA active layer [86]; (**C**) confinement effect [24]; (**D**) thinner PA layer formed on the substrate with larger pores and increased in the possible defects’ effect on the rejection of the TFC membrane [95]; (**E**) hydrophilicity effects of substrate on the formation of PA active layer [22]; (**F**) pattern replication represented by AFM cross-sectional profiles for patterned TFC membranes [99].

The porosity and pore size of the substrate have significant effects on the permeance of TFC. On the one hand, the porosity of the substrate can greatly regulate the water transport pathway [19,82,83]. The cylinder pores model [7] for porous membranes and the solution-diffusion (S-D) model [84] for nonporous membranes are only suitable for free-standing active layers where the solvent transport is in the perpendicular direction. However, for a TFC membrane, the solvent needs to seek ways to enter the discrete pores of the substrate when leaving the top active layer, which results in a longer transport pathway compared to the intrinsic thickness. This phenomenon is referred to as the funnel effect [20,21]. Models indicate that the real permeance (A_real_) could approach the ideal permeance (A_ideal_) when the porosity of the substrate is high enough (approximately 100%), as shown in Figure 3A. Furthermore, the funnel effect is more severe for thinner active layer thicknesses, as the model shows that the larger *l*/R_2_ results in greater scaled permeance [19]. On the other hand, the pore size of the substrate can effectively regulate the structure of the active layer [23,85]. During the process of fabricating the cross-linked PA active layer, substrates with a larger pore size were reported to store greater amounts of amine, and more amine can participate in the reaction [85]. Moreover, larger pore sizes were also found to facilitate the formation of the PA active layer with a rougher surface [86], which increases the permeance [87,88,89]. This phenomenon was explained by a model called the volcano-like conceptual model [22]. In this model, the eruption of amine solution from the pores during the interfacial polymerization results in rougher PA film, as shown in Figure 3B. However, larger pore sizes could also decrease the performance of the TFC membranes [23,24,26,27,90]. The nanovoids formed in the PA active layer are beneficial for solvent transportation [87]. These voids were shown to be produced by an interfacial degassing mechanism, caused by the interfacial degassing of some gases (CO_2_ or other volatile organic solvents) produced during the interfacial polymerization [90,91,92,93]. Substrates with smaller pore sizes were demonstrated to retain more released bubbles for creating nanovoids in the PA active layer, which was referred to as the confinement effect [23,24] (Figure 3C). Another origin of the nanovoids was explained by localized heating produced during the polymerization, where smaller pores can hinder heat transfer and facilitate the formation of a crumpled film [20,94]. In addition, a relatively large pore size of the substrate was reported to lead to the infusion of the active layer material into the pores, resulting in a poor permeance [26,27]. Based on the above discussions, a substrate with high porosity and moderate pore size could realize a TFC with high permeance. Furthermore, the substrate can also affect the rejection of the TFC membranes. Studies have found that a substrate with a very large pore would increase the possible defect formation in the active layer and its effects of defects on rejection [86,95], as shown in Figure 3D. Additionally, the intrinsic properties of the active layer, such as crosslink degree and surface uniformity, can be adjusted by the pore size and porosity of the substrate, which also exerts great effects on rejection [96,97]. Modifying the porosity and pore size of bulk substrate to enhance the fabrication of a superior active layer often entails compromising the filtration performances of the substrate. However, selectively adjusting the pore structures solely on the substrate surface [98] or implementing an interlayer (as discussed in Section 4) becomes possible to preserve the substrate’s performance more effectively.

#### 3.1.2. Hydrophilicity

Generally, a hydrophilic substrate can store a higher content of monomers for interfacial polymerization. A conceptual model proposed by Ghosh and his coworkers [22] illustrated that the aqueous solution meniscuses in the hydrophilic and hydrophobic substrate pores are concave and convex, respectively, and therefore, more polyamide is formed in the pores of a hydrophilic substrate, as shown in Figure 3E. The advantage of using a hydrophilic substrate for PA TFC is exhibiting better substrate/active layer adhesion, but a substrate that is too hydrophilic could lead to a thick PA layer and more penetration of PA in the pores [25]. Additionally, choosing a hydrophobic substrate has limitations due to poor quality layer formation and very low storage of amine monomer on the substrate, which usually exhibits very low rejection [100,101]. The optimum water contact angle values could be between 40° and 60° [102].

#### 3.1.3. Roughness

In general, surface roughness can enhance the hydrophilicity or hydrophobicity of the substrates [103]. Studies have also focused on improving the roughness of the porous substrate by surface patterning techniques [99,104,105]. One such technique is nanoimprint lithography (NIL), which is used to impart sub-micro surface patterns onto the porous substrate [104]. The interfacial polymerized PA layer can typically replicate the pre-formed pattern on the substrate. Such modification can not only improve the real separation area of the active layer but also alleviate the formation of solute concentration polarization on the surface, effectively enhancing the permeance and antifouling performance of the TFC membranes, as shown in Figure 3F.

### 3.2. Substrate’s Effect on LLC-Based Active Layer

Several models and theories that were developed to explain the effects of substrates on PA-based TFC membranes can also be applied to LLC-based TFC membranes. Among these models, the funnel effect and confinement effect suggest that the substrates with high porosity and small pore size may be preferable for supporting the LLC active layer. Moreover, a smaller pore size substrate can prevent the infiltration of LLC mixtures into the pores. However, unlike interfacial polymerization, where amine monomers quickly diffuse from the pores during the reaction, the volcano-like conceptual model may not be suitable for the LLC system, which is typically driven by free-radical photopolymerization [65]. Additionally, the membrane formation mechanism indicates that a flat substrate with suitable hydrophilicity is required to form a uniform and flawless LLC-based active layer. Unlike the PA active layer, which forms membrane structures after interfacial polymerization, the final structure of the LLC active layer is significantly influenced by the formed LLC mesophase before photopolymerization. As the LLC mesophase contact with the substrates before polymerization, this section discusses the substrate’s effects on the LLC mesophase.

Section 2.1.2 shows that the behaviors of the LLC phase, such as phase structure and unit cell dimension, which are determined by both interfacial curvature and hydrocarbon packing energy, can be easily altered by a variety of external conditions. Therefore, the effects of substrate on the LLC phase structure and unit cell size cannot be ignored. Numerous studies have investigated surfactant mesophases formed on solid substrates, and it has been demonstrated that the surface properties of substrates, including hydrophilicity and heterogeneity, can modify the interfacial curvature of surfactant mesophases formed on them [18]. Moreover, Grady et al. [106] directly probed the surfactant mesophases on nanoscopic trenches and pillars using an atomic force microscope. They found that the effect of surface physical heterogeneity on the structure of surfactant mesophases extends to lengths much longer than those of individual surfactant molecules (approximately 50 nm vs. 2 nm). Given that the promising thickness of the LLC active layer is around 200 nm, the phase structure of the LLC can be significantly influenced by the substrate.

#### 3.2.1. Effects of Substrate Hydrophilicity on the Structure of LLC Mesophases

**Figure 4:**
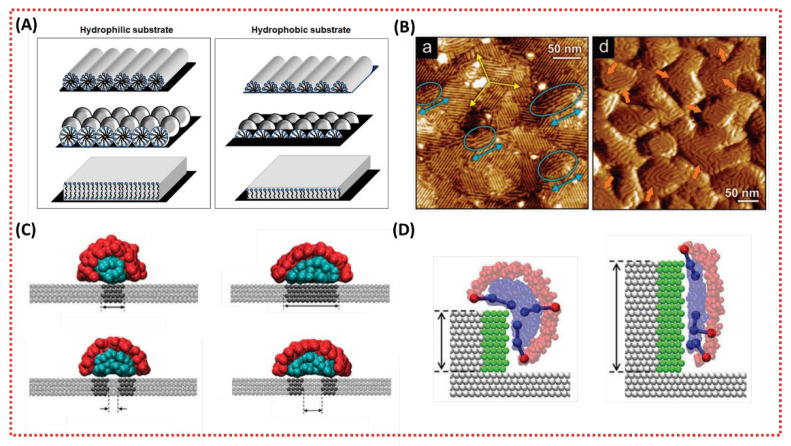
(**A**) Schematic of typical surfactant mesophases formed on substrate with various hydrophilicity. Blue spheres are the hydrophilic surfactant headgroups, and black curves are hydrophobic tail groups [18]; (**B**) AFM images for gold surfaces in contact with SDS solutions at 10 mM concentration. (**a**) Oriented parallel hemicylinders SDS were observed on flat flame-annealed gold surface (yellow arrows highlight the micelles are oriented in one of three preferred orientations on large and flat areas, and blue arrows and ellipses highlight that micelles exhibit orientations in different directions only at topographic steps). (**d**) No orientation order can be observed on the even rougher gold surface (orange arrows indicate areas where the micelles entwine around the grains) [113]; (**C**) surfactant mesophases were absorbed on a flat substrate with hydrophobic stripes. The hydrophobic and hydrophilicity surfaces are shown as dark and light gray, respectively. Surfactant head and tail groups are shown as red and cyan spheres [114]; (**D**) surfactant mesophases were absorbed on steps. The hydrophobic and hydrophilicity surfaces are shown as green and light gray. Surfactant head and tail groups are shown as red and blue spheres [115].

The hydrophilicity of the substrates can significantly alter the structure of LLC mesophases. Several studies have investigated surfactant adsorption at the hydrophilic or hydrophobic solid–water interface [18,107,108,109,110,111]. The relatively weak bond between the substrate surface and solvent causes the surfactants to replace solvent molecules at the surface. Typically, cylindrical, spherical, and bilayer mesophases form on hydrophilic surfaces, whereas hemicylindrical, hemispherical, and monolayer mesophases form on hydrophobic surfaces, as shown in Figure 4A [18]. In hydrophobic systems, the hemi- and mono-behavior is related to lower free energy and minimal contact between the hydrophobic substrate and water [112].

#### 3.2.2. Effects of Substrate Heterogeneity on the Structure of LLC Mesophases

Surface heterogeneity can have a significant impact on surfactant adsorption and phase formation, as reported in previous studies [18,116]. The surface heterogeneity can be categorized as either chemical, physical, or a combination of both. Most computational studies have focused on flat surfaces with chemical heterogeneity [114,117]. Surfactant adsorption is based on the interaction between the hydrophobic tail of surfactant and hydrophobic areas, which can effectively alter the interfacial curvature of formed mesophases through lateral confinement. Surfactant mesophases with higher interfacial curvature can be achieved by forming mesophases on a narrower hydrophobic strip or two closer adjacent strips, as shown in Figure 4B [114,117]. Physical heterogeneity also has significant effects on surfactant mesophases. Sodium dodecyl sulfone (SDS) mesophases with lower interfacial curvature were formed on a rougher gold substrate, as shown in Figure 4C [113]. Furthermore, the interfacial curvature of adsorbed surfactant mesophases decreases with the increasing length of the hydrophobic strip on steps, as shown in Figure 4D [115]. Additionally, the effects of physical heterogeneity on the surfactant mesophases are associated with surfactant concentration [118], electric charge [119], and the existence of co-adsorbents (monomers or crosslinkers) [120].

## 4. Substrate Surface Modification

### 4.1. Ideal Substrate Surface Structure and Properties

In order to achieve high water permeance and rejection in both PA-based and LLC-based TFC membranes, it is important to choose substrates with high porosity (around or above 10%) and small to moderate pore size [19]. For PA-based TFC membranes, substrates with suitable surface hydrophilicity enhance the rejection properties and improve adhesion between the top layer and substrate without decreasing water permeance. In contrast, substrates with suitable surface heterogeneity provide higher antifouling ability and a more effective separation area. Regarding LLC-based TFC membranes, the surface properties of the substrate play a crucial role in achieving a uniform and thin LLC active layer, regardless of the LLC phase structure. Specifically, substrates with higher surface hydrophilicity and lower surface roughness are preferred. On the other hand, LLC active layer with a suitable phase structure and unit cell dimension can be achieved by controlling the interfacial curvature of LLC mesophase, which can be accomplished by altering the surface hydrophilicity and heterogeneity of the substrate.

### 4.2. Surface Modification

Porous substrates, such as MF and UF membranes, which are commonly prepared by the phase inversion method, are used to support the active layer to build thin-film composites. However, controlling the structure formation of such substrates is challenging due to multiple factors during the phase inverse process. UF membranes usually have smooth and dense surfaces but are limited by their low porosity (<10%), while MF membranes usually have oversized porous structures that decrease the stability of the active layer [121,122]. Moreover, polymeric porous membranes are often not hydrophilic enough, leading to an uneven distribution of monomer solvent on the substrate surface and an unstable interface between the active layer and the substrate, which may result in potential detachment. Therefore, finding suitable surface modification strategies is crucial to design the interfacial surface between LLC active layer and substrate. The main motivation of the reported surface modification techniques is to enhance the hydrophilicity of membranes and thus improve their antifouling performance [29]. For LLC thin-film composite membranes, modified substrates with high hydrophilicity and smooth surface are promising. Two surface modification strategies can help us obtain substrates with ideal surface properties:

- Surface deposition;
- Surface grafting.

#### 4.2.1. Surface Deposition

**Figure 5:**
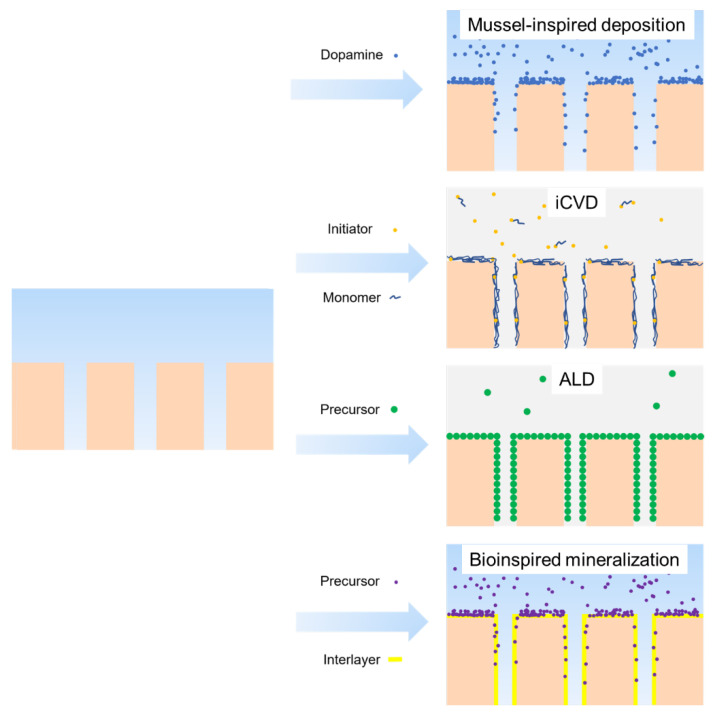
Emerging strategies of surface deposition.

Deposition methods can be categorized into mussel-inspired deposition, atomic layer deposition, initiated chemical vapor deposition, and bioinspired mineralization (Figure 5).

Among these methods, mussel-inspired deposition has gained popularity in recent years, and dopamine and polyphenol tannic acid are two common materials for this purpose. These materials are inspired by the 3,4-dihydroxyphenylalanine found in mussel food proteins and can be oxidized and self-polymerized to create a uniform coating on polymer materials, enhancing the hydrophilicity of the membrane surface [29]. The detailed reaction mechanism can be found in previous literature [123,124]. The widespread use of this deposition method is due to the following reasons:

- This modification occurs in mild and wet conditions, which prevents membrane pore collapse during drying [125] and avoids degradation caused by some other methods, such as plasma or irradiation methods [126];
- No special reaction is required between the deposition layer and membrane surface since the deposition material interacts with hydrophobic and hydrophilic membranes through π–π stacking/hydrophobic interactions and electrostatic/hydrogen bonds/covalent bonds interaction, respectively [127,128];
- The coating layer thickness can be easily adjusted by varying the deposition time and solvent concentration, which prevents pore size blocking, especially for UF [129];
- Further functionalization can be achieved since the deposition layer introduces amino and hydroxyl groups onto the membrane surface [130].

The three main disadvantages of the deposition method are long time consumption, unstable coating, and environmental pollution caused by suspended deposition particles. However, emerging methods such as co-deposition [131,132,133], introducing oxidants [134,135], and applying irradiation [136,137] have been developed to improve deposition efficiency and stability.

Compared to mussel-induced deposition, initiated chemical vapor deposition (iCVD) is better at forming a uniform organic coating on various substrates. During the iCVD process, the substrate is exposed to a volatile initiator and one or two vinyl monomers, on which the initiator is decomposed to radicals and initiates the radical polymerization and creates organic coating in one step [138]. This method is easy to use to fabricate homopolymer or copolymer coatings in nanometer thickness on polymer substrates. The substrate’s surface can be kept at a relatively low temperature (<40 °C) [138] during the process because the initiator is decomposed by a resistively heated filament array, which makes it possible to coat some polymers such as PTFE that requires a high-temperature sintering step (400 °C) [139]. Since this process occurs at the gas–solid interface and in a vacuum chamber, membranes with nanopores sensitive to capillary forces during drying are not suitable to be treated [125]. Conversely, this solvent-free method can effectively avoid the potential damage brought by the solvent to the membrane, such as swelling, acid corrosion, and shrinkage [140].

Compared to forming organic coatings by mussel-inspired deposition and iCVD, atomic layer deposition (ALD) is better at forming nanosized conformal inorganic coatings on various polymer substrates, which is also a vapor-phase (inorganic precursor vapors) conformal deposition process occurring at the solid–gas interface [141]. However, ALD is an alternate deposition method rather than a one-step deposition method. This method can form more uniform coatings on polymers with polar surface since they provide enough nucleation site, while form discrete particles on nonpolar polymers [142]. The method is limited by the vapor pressure of the precursors, as many precursors need high temperature to be vaporized. This can reach the melting point of polymers, which is why Al_2_O_3_, TiO_2_, and ZnO, which possess precursors with high vapor temperature at low temperature, are the most commonly used inorganics in ALD [143].

Another strategy to deposit an inorganic coating on the membrane surface is bioinspired mineralization. In this process, an interlayer (e.g., poly (acryl acid) or PDA/PEI) needs to be pre-created on the hydrophobic membranes. Subsequently, carboxyl-induced calcification, amino-induced silicification, or catechol groups-induced metal ions chelation will form corresponding CaCO_3_ [144], SiO_2_ [145], TiO_2_ [146], or ZrO_2_ [147] mineral coating. Compared to the ALD, this method can be conducted at ambient temperature and does not depend on the equipment.

#### 4.2.2. Surface Grafting

**Figure 6:**
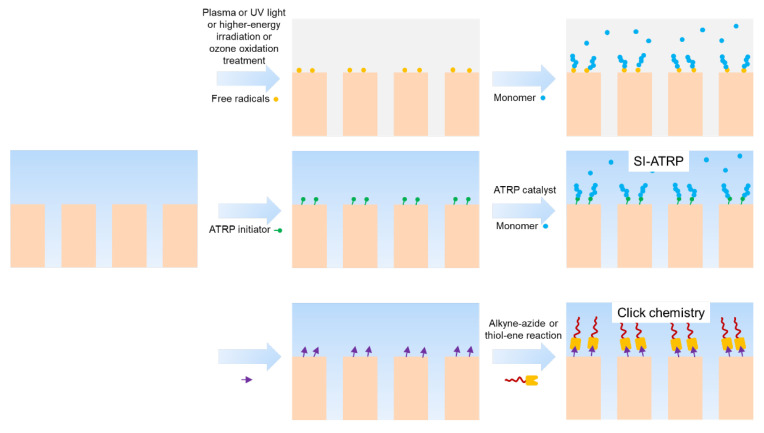
Emerging strategies of surface grafting.

Surface grafting involves covalently linking polymer chains onto the membrane surface This process can be achieved through two strategies: “grafting to” and “grafting from” (Figure 6). Compared to the “grafting to” method, the “grafting from” method is more attractive due to its high grafting density. Surface grafting techniques can be categorized as either chemical or physical. Physical techniques involve grafting induced by plasmas, UV light, and high-energy irradiation. Chemical techniques include natively initiating, surface-initiated atom transfer radical polymerization (SI-ATRP), click chemistry, and ozone oxidation (Table 2).

### 4.3. Interlayer

**Figure 7:**
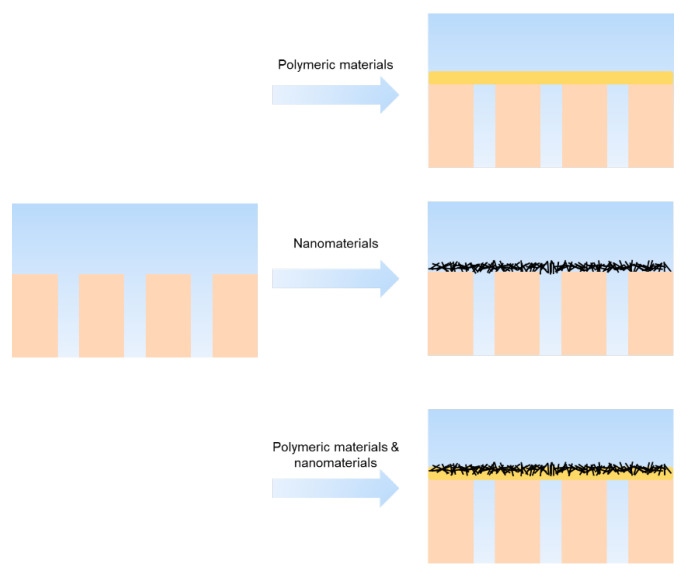
Constructing interlayers with various materials.

In addition to the surface modification mentioned above, introducing an appropriate interlayer on MF or UF membranes offers a method for manipulating the porous structure and other surface properties of the substrate. This assists with active layer formation and improves the performance of TFC membranes. Interlayer materials can be divided into polymeric materials and nanomaterials (Figure 7). Various methods have been developed for fabricating interlayers, including dip-coating, vacuum filtration, co-deposition, in situ synthesis, spin-coating, electrospray-coating, spray-coating, and brush-coating [168].

#### 4.3.1. Polymeric Interlayer

Polydopamine possesses suitable reactivity and stability driven by both covalent and non-covalent interactions, making it promising for forming interlayers. The co-depositing method has been used to fabricate interlayers by combining dopamine with other amine-rich polymers, such as PEI, to suppress self-polymerization [169]. Additionally, the CuSO_4_/H_2_O_2_-triggered method has been used to improve the reaction rate [133,170,171,172]. Beyond PDA-based polymeric materials, Polyvinyl alcohol (PVA) [173,174] and tannic acid (TA) [31,175,176] have gained attention due to their being more cost-effective and environmentally friendly. These interlayers are commonly dip-coated and co-deposited on porous substrates, are usually hydrophilic, and improve compatibility between the substrate and active layer material. This improves the homogeneous dispersion of the pre-polymerization solvent, forming a more uniform and stable active layer [121]. Polymeric-based interlayers are commonly more environmentally friendly compared to the nanomaterials, and TFCs based on polymeric interlayers are more robust with stronger adhesion force and higher rejection properties than that of the one based on nanomaterials interlayers.

#### 4.3.2. Nanomaterial as Interlayer

Nanomaterials can be differentiated by various dimensions. One-dimensional (1D) nanomaterials, such as Cd(OH)_2_ nano-strand [20], covalent organic framework (COF) nanofiber (high porous structure, low density, and cationic feature) [177], carbon nanotubes (usually coated with PDA) [168,178,179,180,181], and cellulose nanocrystal (CNC) (non-toxic and environmental friendly) [182] and 2D nanomaterials, such as graphene oxide (GO) [183], covalent organic framework (COF) nanosheets [184], and MXene [185], are commonly vacuum filtrated, sprayed, or brushed onto the porous substrate to form a uniform and continuous interlayer with closely packed structure. However, 3D nanomaterials, such as metal organic framework (MOF) [186,187] materials, cannot use the vacuum filtration method to form interlayers directly due to their less uniform steric configurations. In this case, new methods such as in situ synthesis [187] and Langmuir–Schaefer (LS) [186] have been developed to form a continuous MOF interlayer. Compared to polymeric interlayer-based TFCs, those based on nanomaterials possess higher permeance.

#### 4.3.3. The Effect of Interlayer on the Interface of Interlayer/Substrate and Interlayer/Active Layer

The introduction of an interlayer alters the interface between the interlayer and substrate, as well as interlayer and active layer. On the one hand, the interlayer/substrate interface plays a significant role in the adhesion forces between them. For instance, when a polymeric interlayer is dip-coated onto a porous substrate, the infusion of polymers into the pores is inevitable due to the possible interactions between them, such as hydrogen bonds, electrostatic interactions, van der Waals forces, and π–π conjugation. This improves the adhesion forces between the interlayer and substrate but can lead to permeability loss. Therefore, it is crucial to choose a coating solution with suitable viscosity to prevent excessive penetration. Moreover, nanomaterials typically form interlayers with the aid of polymer materials since they cannot adhere firmly to the substrate due to the limited contact area. On the other hand, the interlayer/active layer interface significantly affects the thickness and structure formation of the active layer.

I. Reducing the surface pore size and roughness and increasing surface hydrophilicity can improve the dispersion of the pre-polymerization solvent, resulting in an active layer with minimal defects.
II. Enhancing surface wettability and altering surface charge can control the diffusion of monomers and adjust the thickness and crosslinking degree of the active layer [31].
III. Introducing an interlayer can improve the confinement effect for interfacial degassed nanobubbles, increasing the surface roughness of the active layer, and enhancing permeability.
IV. Functional groups on the interlayer can participate in the formation reaction of the active layer, and thereby improving membrane rejection.
V. Overall permeability can be improved by shortening the water path in the less-permeable active layer and increasing the water path in a more permeable interlayer [30].

#### 4.3.4. Machine Learning for Interlayer Material Choose and Prediction of Modified Surface Performance

There is a plethora of materials available for constructing the interlayer, each exhibiting unique performance characteristics and dimensions. Therefore, the task of identifying the most optimal interlayer material and maximizing the surface performance of the substrate proves to be a challenging endeavor. Artificial intelligence technologies are assuming ever-expanding roles in the domains of engineering design and scientific research. Machine learning has the capability to extend the scope of tasks, thereby offering promising prospects for advancing modeling techniques in system prediction and optimization [188]. The general steps of using machine learning for selecting a suitable interlayer material for surface modification and predicting surface properties could follow these general steps:

1. Data preparation: Gathering a dataset that includes information about various interlayer materials, their properties, and the corresponding surface modifications process and substrate surface properties. These data should cover a diverse range of materials and surface characteristics.
2. Data preprocessing and feature engineering: Cleaning and preprocessing the collected data. This involves handling missing values, normalizing or scaling the data, and encoding categorical variables if necessary, then extracting relevant features from the dataset that can effectively capture the characteristics of interlayer materials and their impact on surface properties.
3. Model training: Selecting an appropriate machine learning algorithm, such as regression and classification, depending on the specific prediction task. Splitting the dataset into training and testing sets and training the model using the training data.
4. Model evaluation: Evaluating the trained model’s performance using the testing dataset. Using appropriate evaluation metrics.
5. Predictions and model optimization: Once the model is trained and evaluated, utilizing it to make predictions on new, unseen data. Inputting the relevant features of an interlayer material and modification process and the model will provide predictions for substrate surface properties. Then, fine-tuning and optimizing the machine learning model to enhance its predictive accuracy. This may involve a hyperparameter tuning model.

## 5. Characterization of LLC Phase/Substrate Interface

**Figure 8:**
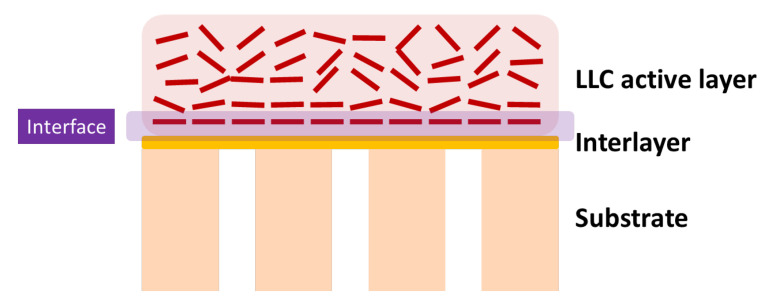
Cross-sectional surface of TFC based on LLC active layer.

Effective characterization methods need to be developed for studying the substrate’s effect on LLC phase structure and thus better facilitating the substrate’s control over the LLC active layer structure. Promising techniques are described as follows for studying the interface between LLC and the substrate (Figure 8).

### 5.1. X-ray and Neutron Techniques for the Characterization of the Structures of LLC Films

#### 5.1.1. Small-Angle Scattering Technique

Elastic scattering produces an interference phenomenon and therefore carries the structure information of LLCs. Small-angle scattering (SAS) techniques can capture the elastic scattering information of LLCs. The actual size of the lattice or object determines the chosen light source with a specific wavelength. Small-angle (2*θ* < 5°) X-ray/neutron scattering (SAXS/SANS) is the optimal tool to study objects in the size range of 1–100 nm since the *q*-range related wavelength of X-ray and neutron covers this range. The incidence X-ray wave interacts with electrons for X-ray scattering, while the scattering occurs between the incidence neutron and nucleus for neutron scattering. The contrasts of X-ray and neutron scattering are from electron density and nuclear scattering length density (SLD), respectively. The diffraction pattern of elastic scattering can be exhibited by a *q*-vector. The relationship between the incidence angle and *q*-vector can be presented by [189]:

(3) $$q=\frac{4\pi \sin\theta }{\lambda }$$

where *λ* is the wavelength of the light source, and *θ* is half of the angle between the incidence wave and the scattering wave. *λ* is fixed for the common mode while the *θ* is fixed and *λ* is variant for the time-of-flight (TOF) mode [32].

The distance between repeated planes in the lattice of matter is the most important structure factor to be studied. The relationship between the distance *d_hkl_* and scattering angle can be given by [189]:

(4) $$n\lambda =2d_{hkl}\sin\theta$$

where *n* is the reflection order.

Therefore, the relationship between the *d* and *q*-vector can be given by:

(5) $$d_{Bragg}=\frac{2\pi }{q_{Peak}}$$

#### 5.1.2. Small-Angle X-ray Scattering

SAXS is the most direct tool to identify the LLC mesophases before and after UV-curing. In addition to phase identification, SAXS can also provide accurate spatial information, including size, shape, and arrangement of the nanostructure. Foudazi et al. have summarized multiple structural parameters of LLC mesophases that can be calculated from the position of Bragg peaks [17].

#### 5.1.3. Small-Angle Neutron Scattering

SANS has three special features that make it complementary to SAXS:

1. Neutron is neutrally charged and non-destructive to costly samples, and they can deeply penetrate into the atom to interact directly with nuclei;
2. The scattering power of neutrons is not related to the number of atoms, making lighter elements such as hydrogen more distinctive;
3. Sample contrast can be altered to suit specific needs by partial deuteration.

The contrast of scattering length density (SLD) can be enhanced by the isotope in neutron scattering, which makes various ingredients in the sample more distinctive. This technology is known as contrast variation. The *SLD* can be given by [190]:

(6) $$SLD=\frac{\rho N_{a}\sum_{i=1}^{N}b_{i}}{\sum_{i=1}^{N}M_{i}}$$

where *ρ*, *N_α_*, *M_i_*, and *b_i_* are the bulk density of the molecule, the Avogadro constant, the atomic molar mass for each element, and the scattering length contribution from *N* atoms with the unit cell or molecule, respectively.

Moreover, neutron scattering has the ability to detect the exact location of movable monomers and crosslinkers within the lyotropic liquid unit cell [191,192,193]. Leonie et al. reported that the lipid LLC phases can be contrast-matched, and the scattering from encapsulated peptides and proteins can be isolated. This enables them to better understand the location of the desired molecules during LLC structure evolution and phase transition [191,192]. Since the locations of monomers and crosslinkers during polymerization directly determine the pore structure of the as-synthesized LLC-templated materials, and the locations of these molecules could be very different at LLC/substrate interface, contrast matching the deuterated amphiphile molecules with solvents (a mixture of d-solvent and h-solvent) makes it possible to better detect and control the interface structures.

#### 5.1.4. Grazing Incidence Small-Angle Scattering (GISAS)

**Figure 9:**
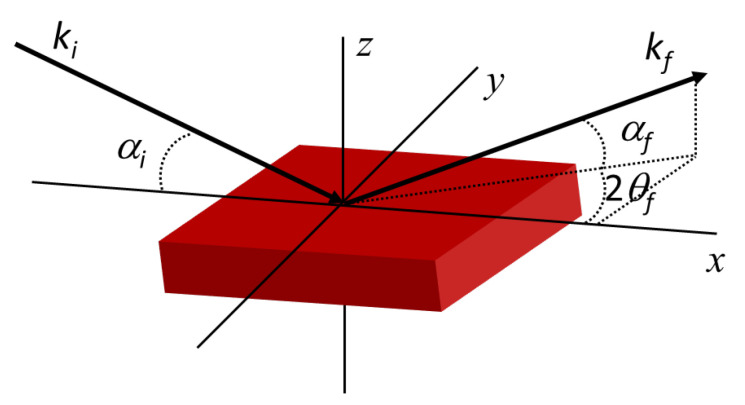
Geometry schematic of specular reflection and GISAS experiments.

Bulk samples of LLC before and after UV-curing can be studied very well using transmission geometry techniques, such as SAXS and SANS, while reflection geometry using grazing angles is necessary for studying thin-film samples with a reduced scattering volume. GISAS studies the surface or interface of film nanostructures under grazing incidence geometry. Specular reflectivity of the incident beam occurs when the film is absolutely smooth, while off-specular reflectivity occurs when the film is rough and inhomogeneous. Figure 9 is the schematic geometry of specular and GISAS experiments. *xy* is the sample surface plane. *x* axis is the projection of incidence beam with an incident angle of *α_i_* on the sample surface. *α_f_* and 2*θ_f_* are the exit angle and out-of-plane angle, respectively. *k_i_* and *k_f_* are the wavevector of incident and exit beam, respectively. The wave vector transfer can be given by *q* − *k_f_*-*k_i_*. For specular reflection, *α_f_* = *α_i_* and 2*θ_f_* = 0, so *q_x_* = *q_y_* = 0 and only *q_z_* > 0. In this case, the scattering vector *q* = *q_z_* = 4πsin*α_i_*/*λ*.

For off-specular scattering (α*_f_* ≠ α*_i_* and 2*θ_f_* ≠ 0), the components of the scattering vector can be given as [32,194]:

(7) $$\begin{array}{c} q_{x}=\frac{2\pi }{\lambda }(\cos\alpha_{f}\cos{2\theta }_{f}-\cos\alpha_{i})\\ q_{y}=\frac{2\pi }{\lambda }(\cos\alpha_{f}\sin{2\theta }_{f})\\ q_{z}=\frac{2\pi }{\lambda }(\sin{2\theta }_{f}+\sin\alpha_{f}) \end{array}$$

The 2D detector mainly probes the information of *q_z_* and *q_y_*, since *q_z_*, *q_y_* >> *q_x_*. The scattering intensity of *I*(*q_y_*, *q_z_*) is commonly detected for studying films’ structure in the *y* axis and *z* axis.

The intensity of the wave scattering from the LLC top layer can be given by [195,196,197]:

(8) $$I_{GISXS}=\frac{d\sigma }{d\Omega }=A\frac{\pi^{2}}{\lambda^{4}}{\left(1-n^{2}\right)}^{2}{\left|T(k_{i})\right|}^{2}{\left|T\left(k_{f}\right)\right|}^{2}F(q)$$

where d*σ*/d*Ω* is the differential cross-section of the film, which is expressed by the framework of the distorted-wave Born approximation (DWBA). *A* is the illuminated area. *T*(*k*) is the Fresnel transition function, whose maximum corresponds to the Yoneda peak of the differential scattering when the incident angle *α_i_* equals the critical angle *α_c_* of LLC film. *n* is the refractive index of the beam in the medium, which can be given by [198]:

(9) n=1−δ+iβ

where *δ* and *β* are the dispersion and absorption of the beam inside the medium, respectively.

In the case of GISAXS, the dispersion can be given by [198]:

(10) $$\delta_{x}=\frac{\lambda^{2}\rho_{el}r_{0}}{2\pi }$$

where the *ρ_el_* and *r*_0_ are the electron density and the classical electron radius (2.82 × 10^−13^ cm), respectively.

The absorption can be given by:

(11) $$\beta_{x}=\frac{\mu \lambda }{4\pi }$$

where *μ* is the linear absorption coefficient.

In the case of GISANS, the dispersion can be given by [32]:

(12) $$\delta_{N}=\frac{\lambda^{2}SLD}{2\pi }$$

The absorption can be given by:

(13) $$\beta_{N}=\frac{N\alpha_{a}\lambda }{4\pi }$$

where *N* and *α_a_* are the atomic number density and absorption cross-section for neutrons, respectively. The *β_N_* is not necessary to consider in most cases.

The *n* and *T* act only as overall scaling factors since *α_i_* and *α_f_* are fixed for GISAS experiments. The detected scattering intensity is mainly determined by the diffuse scattering factor *F*(*q*). For *N* identical and centro-symmetrical objects with a random orientation in the LLC system, the *F*(*q*) can be approximated by:

(14) $$F\left(q\right)\sim NS(q)P(q)$$

where *S*(*q*) and *P*(*q*) are the structure and form factors of individual objects, respectively. The *S*(*q*) yields information on object positions, such as the interparticle distance, while the *P*(*q*) provides information about the shape and size of the objects [195,196].

The scattering depth is another essential point for GISAS, which is given by [32,33,199]:

(15) $$D=\frac{\lambda }{\sqrt{2}\pi \left(l_{i}+l_{f}\right)}$$

where the *l_i_* and *l_f_* depend on the incident angle and exit angle of the beam, and absorption and critical angle of the medium.

For X-rays:

(16) $$l_{i,f}={\left[{\sin\alpha_{c}}^{2}{-\sin\alpha_{i,f}}^{2}+\sqrt{{\left({\sin\alpha_{i,f}}^{2}{-\sin\alpha_{c}}^{2}\right)}^{2}+{\left(\frac{\mu \lambda }{2\pi }\right)}^{2}}\right]}^{1/2}$$

For neutrons:

(17) $$l_{i,f}={\left[{\sin\alpha_{c}}^{2}{-\sin\alpha_{i,f}}^{2}+\sqrt{{\left({\sin\alpha_{i,f}}^{2}{-\sin\alpha_{c}}^{2}\right)}^{2}+{\left(\frac{N\alpha_{a}\lambda }{2\pi }\right)}^{2}}\right]}^{1/2}$$

**Figure 10:**
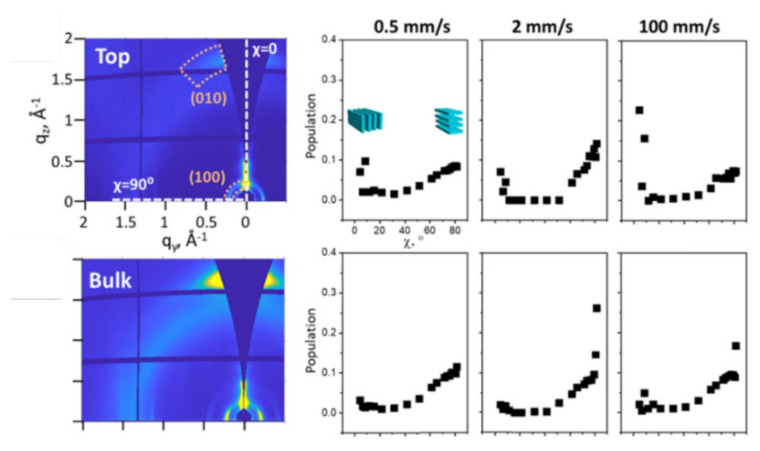
Two-dimensional (2D) grazing incidence wide-angle X-ray scattering patterns for top interface and bulk and corresponding partial pore figure of conjugated polymer film [200]. The above speed values are film coat speeds.

In general, neutrons have a higher scattering depth than X-rays due to their lower absorption by the medium. However, the flux limitation of GISANS can result in smearing in scattering depth, while GISAXS typically has a high wavelength resolution. For both beams, it is possible to obtain both near-surface and bulk LLC mesophase structures by measuring at α_i_ < α_c_ and α_i_ > α_c_ (Figure 10). Generally, the thickness of the active layer is around 200 nm. The interfacial structure can be identified by comparing the near-surface and bulk LLC structure of this thin layer.

#### 5.1.5. X-ray and Neutron Reflectivity

**Figure 11:**
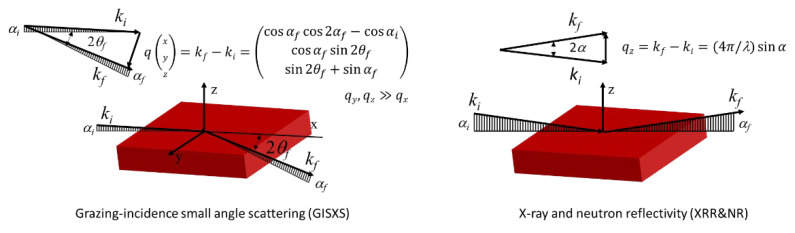
Schematic of horizontal (GI) mode and vertical (reflectivity) mode [201].

**Figure 12:**
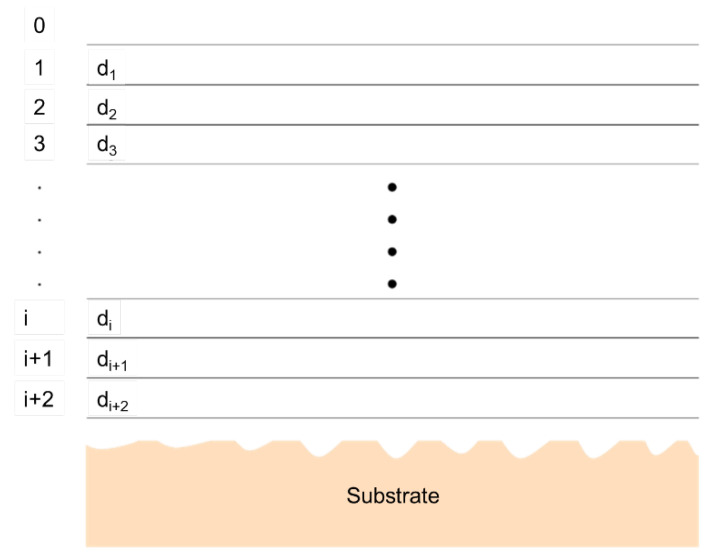
Diagram of an LLC film comprises of i + 2 layers with a variable thickness on a substrate.

GISAS is sensitive to the lateral structure of the LLC active layer, while X-ray and neutron reflectivity can be used to study the structural properties along the normal surface (Figure 11). The evolution of the LLC active layer structure can be assumed to comprise different LLC layers of varying thicknesses (Figure 12), and reflectivity techniques can be used to detect the thickness of each layer. In specular reflectivity, the angle of incidence and departure are equal, so only the z component of momentum transfer (normal to a surface) is of interest, which is given by [198]:

(18) $$k_{z,0}=(2\pi /n)\sin\theta$$

where *λ* and *θ* are the wavelengths of the incident beam and the angle of incidence, respectively. The subscript 0 represents vacuum. For the LLC top layer 1, the z component of the momentum transfer is given by:

(19) $$k_{z,1}=\sqrt{k_{z,0}^{2}-4\pi \rho }$$

where *ρ* is the electron density for X-ray or scattering length density for the neutron of the layer. 

For the air or vacuum interface with LLC top layer, the reflection coefficient can be given by:

(20) $$r_{0,1}=(k_{z,0}-k_{z,1})/(k_{z,0}+k_{z,1})$$

The reflection coefficient *r_i,r_*_+1_ of the two separated LLC layers *i* and *i* + 1 can be given by:

(21) $$r_{i,i+1}=(k_{z,i}-k_{z,i+1})/(k_{z,i}+k_{z,i+1})$$

The connection between *r* and layer thickness *d* can be given by:

(22) $$r_{0,1}=\frac{r_{0,1}+r_{1,2}exp(2id_{1}k_{z,1})}{1+r_{0,1}r_{1,2}exp(2id_{1}k_{z,1})}$$

The reflection coefficient *r_i,i_*_+1_ can be given by:

(23) $$r_{i,i+1}=\frac{r_{i,i+1}+r_{i+1,i+2}exp(2id_{i+1}k_{z,i+1})}{1+r_{i,i+1}r_{i+1,i+2}exp(2id_{i+1}k_{z,i+1})}$$

The reflectance at the air/LLC film interface *r*_0,1_ can be calculated by following the recursion through the *i* interfacial layer adjacent to the substrate.

The reflectivity *R* is given by:

(24) $$R\left(k_{z,0}\right)=1\;(k_{z,0}\leq k_{c,1})$$

(25) $$R(k_{z,0})=\frac{r_{0,1}^{2}+r_{1,2}^{2}+2r_{0,1}r_{1,2}\cos\left(2k_{z,1}d\right)}{1+r_{0,1}^{2}r_{1,2}^{2}+2r_{0,1}r_{1,2}\cos\left(2k_{z,1}d\right)}(k_{z,0}>k_{c,1})$$

Generally, the thickness of the interface layer can be approximated by the separation distance of the minima (Δ*k_z,_*_0_) in the reflectivity profile, *d* = π/Δ*k_z,_*_0_.

**Figure 13:**
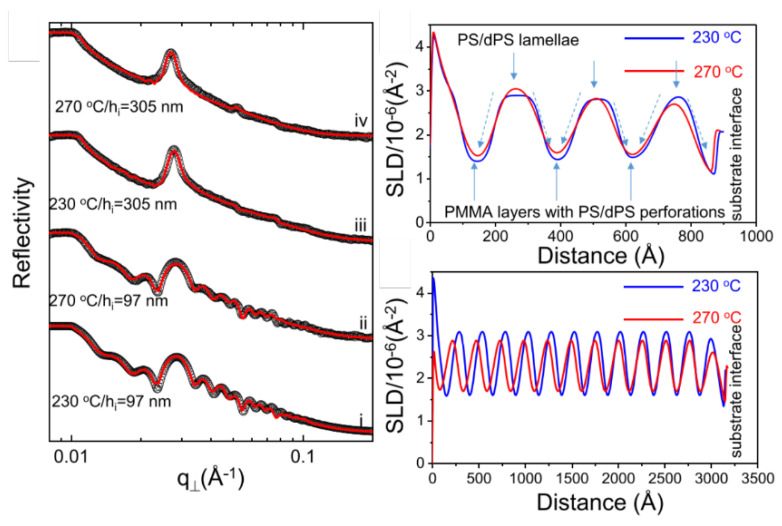
Neutron reflectivity curves and depth-dependent variations in scattering length density for P(S-b-MMA)/dPS blend films [202]. i–iv label the films in various thicknesses and annealing temperatures.

When $k_{z,0}\gg k_{c,1}$, an alternative method has been used to analyze the reflectivity data. In this method, theoretical reflectivity can be expressed as [201]:

(26) $$R(k_{z,0})=\frac{k_{c}^{4}}{k_{z,0}^{4}}{\left|\int \rho (z)exp(2ik_{z,0}z)dz\right|}^{2}$$

where *ρ*(z) is the normalized electron or scattering length density gradient along the z depth in the LLC active layer. $k_{c}^{4}{/k}_{z,0}^{4}$ is the Fresnel reflectivity *R_F_* (*k_z_*_,0_) of an LLC layer with an average scattering length density with an infinitely sharp interface. Therefore,

(27) $$\frac{R(k_{z,0})}{R_{F}(k_{z,0})}={\left|\int \rho (z)exp(2ik_{z,0}z)dz\right|}^{2}$$

where the Fresnel normalized reflectivity is the Fourier transform of the electron or scattering length density gradient. In applying this equation, one can approximate the density gradient along the z depth by fitting a model (Figure 13) [201].

#### 5.1.6. SAS Application in LLC Template Film

**Figure 14:**
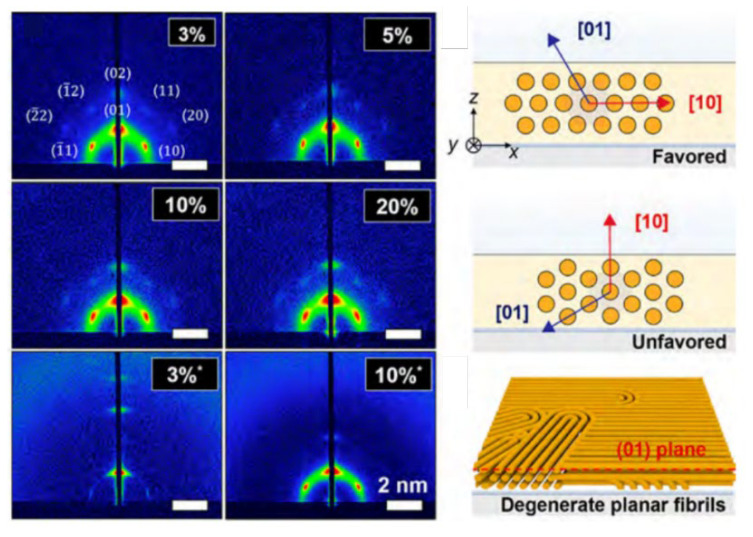
Two-dimensional GISAXS patterns for H_I_ thin film casted on silicon wafer [10].

Powder X-ray diffraction was used to roughly identify the phase structures of LLC template film [16,34]. Afterward, 2D SAXS is commonly used to study the oriented bulk HLLC template films [12,47,203]. The 2D SAXS pattern will vary significantly when altering the incident angle direction of the X-ray beam on oriented LLC films, which identifies the orientation of hexagonal cylinders by analyzing the azimuthal distribution of scattered intensity. A uniform distribution of scattered intensity indicates that the alignment of the hexagonal is random along the incident direction. In contrast, a non-uniform azimuthal intensity means the alignment of the hexagonal cylinder is anisotropic [203]. Osuji et al. [10] used 2D GISAXS to investigate the in-plane morphology in the HLLC films. In this study, they found that the long axes of the hexagonal cylinders lie in the x–y plane by analyzing the intensity of specular and off-specular spots (Figure 14).

### 5.2. Deuterium NMR Spectroscopy

**Figure 15:**
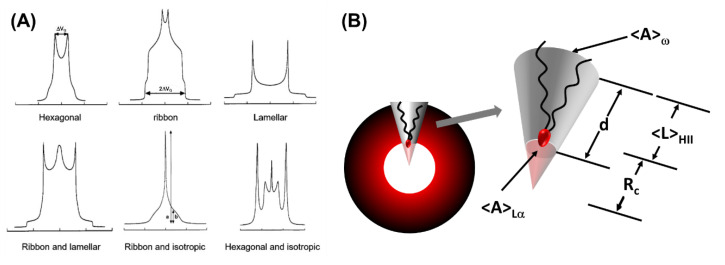
(**A**) Deuterium NMR line shapes of lyotropic liquid crystalline phases of cesium or sodium n-tetradecanoate-D_2_O [205]; (**B**) cross-sectional surface of the reverse hexagonal (H_II_) phase where the average lipid shape corresponds to a frustum of a right circular cone.

Deuterium NMR can provide in-depth structural and dynamic information on lyotropic liquid crystals by detecting the anisotropic mobility and corresponding symmetry of individual surfactant molecules [204]. Deuterium can be isotopically replaced as a probe in hydrogenated surfactant chains, and various lyotropic phases can correspond to characteristic NMR line shapes. The distinctive differences in the LLC phases’ NMR line shape make it possible to distinguish individual phase spectra in a multiple-phase sample (Figure 15A) [205]. Additionally, deuterium NMR can detect the unit cell size of the LLC phase. The unit cell size of the L_α_ phase can be evaluated by the average length of the carbon tail of the surfactant. The average length of the carbon tail in surfactant can be deduced from segmental order parameters, which can be directly obtained from the quadrupolar splitting. The average shape of a surfactant in the H_II_ phase can be approximated as a frustum, as shown in Figure 15B. One can use the cone height *d* to evaluate the unit cell size [206]. Furthermore, the quenching method used in NMR techniques to probe the evolution of crystallization can also be used to retain the phase structure of LLC after being separated from the substrate [207].

## 6. Conclusions and Future Perspectives

This review highlights the interfacial design between LLC and substrate regarding the fabrication of LLC template-based TFC membranes, a comparison of the substrate’s effect on the active layer structure formation and water filtration performances of both LLC- and PA-based TFC membranes, the introduction of suitable modification methods and interlayers, and techniques for interfacial characterization. In general, synergistic templating based on polymerizable amphiphiles is easier to retain LLC template structures during polymerization than the transcriptive method for the construction of LLC template-based TFC membranes. TFC with the best rejection properties has been achieved by an active layer based on a cubic phase with the synergistic templating method, while the highest permeance has been achieved by an active layer based on a parallel-oriented hexagonal phase with the synergistic templating method. Substrates with high porosity (>10%) and small to moderate pore sizes result in high TFC permeance, while a substrate with higher surface hydrophilicity and lower roughness leads to the formation of a more uniform and thinner LLC active layer on it. Several surface modification methods are introduced for improving surface hydrophilicity, while the introduction of an interlayer could control the interface structure formation (e.g., pore size) and shorten the water path. For interfacial characterization techniques, GISAXS is the most direct method to identify the LLC phase structure at the LLC/substrate interface before and after polymerization, while GISANS possesses the ability to localize the monomers at the interface.

Despite some progress that has been achieved in the last 10 years, LLC template TFCs are still in their infancy stage. Significant efforts need to be taken to understand and facilitate the substrates’ effect on the LLC phase, as discussed above. Moreover, the following concerns need to be addressed.

1. Substrate’s effects on LLC phase structures: Although many studies have reported the potential effects of solid substrates on the types and shapes of surfactant mesophases, there has not been a comprehensive analysis of the LLC mesophase structures before and after polymerization at the LLC/substrate interface using SAXS and SANS techniques. To construct GI-mode measurements, ultrathin and uniform LLC membranes must be formed on substrates with varying surface hydrophilicity and roughness, and suitable conditions must be established (e.g., choosing a suitable beam incidence angle to detect the interfacial structures). To conduct neutron measurements, appropriate deuterated ingredients must be introduced to increase the system contrast;
2. Structure retention of LLC parental structure on various substrates (substrate’s effect on polymerization): The retention of LLC parental structure is crucial to avoid phase separation, especially for the transcriptive templating system, and can be achieved by strong interaction between the surfactant template and polymers, as well as timely increase system viscosity and entanglement of polymer chains. In an individual system, the former factor is not easily affected by small changes in interfacial curvature induced by substrates, but changes can occur in the latter factor, which is determined by the polymerization rate. An increase in polymerization rate leads to an increase in the system’s viscosity and chains’ entanglement, which can counterbalance phase separation caused by the increase in free energy. However, the polymerization kinetics are determined by the segregation and diffusion behavior of monomers and initiators during polymerization. The curvature variation induced by substrates changes the LLC order, and for hydrophilic monomers, the polymerization rate increases with an increase in LLC order, while the rate decreases for hydrophobic monomers. In addition, the efficiency of a hydrophilic initiator decreases with an increase in LLC order, whereas it is the opposite for a hydrophobic initiator. Therefore, the substrate is indicated to influence the structure retention of LLC parental structure during polymerization, and a systematic study of this will undoubtedly help to fabricate the high-fidelity LLC-based TFC membranes;
3. Substrate’s effect on reorientation: The substrate’s effect on the reorientation process of LLC cannot be ignored. Achieving less tortuosity in inner porous structures and high permeance of HLLC template membranes is possible through reorientation. However, the HLLC phase typically needs to be heated to a liquid-like isotropic phase during the reorientation process under a magnetic field and electric field, which causes the infusion of the LLC in the substrate. The surface properties of the substrate can interfere with the formation of the HLLC phase during this process. Therefore, designing the surface properties of the substrate suitably and studying the phase formation process in situ during the reorientation is essential for exploring LLC active layers with pores perpendicular to the surface;
4. Substrate’s effect on mechanical properties: The mechanical properties of the LLC template material are significantly associated with the defects and durability of the membrane, which are determined by the width of the continuous phase and the crosslink density of the system. Curvature variation can lead to a wider continuous phase and decrease the crosslink density. Additionally, the substrate’s effect becomes more significant when the active layer’s thickness decreases. Therefore, to fabricate TFC with an ultrathin and flawless LLC active layer, it is essential to address the issue of maintaining or improving the robustness of the LLC template material.

## Figures and Tables

**Table 1:** Summary of the reported results for TFC membranes based on LLC template active layer.

Ref.	LLC Phase	Porous Substrate	Fabrication Process	Solvent	Reactive Temp (°C)	Pore Size in Diameter (nm)	Thickness	Pure Water Flux	Rejection (%)
[34]	H_II_	PSf MF	Roll-casting	H_2_O	RT	1.2	0.6 μm	0.053 Lm^−2^ h^−1^ bar^−1^ μm	Na-TSO (60) Na-NpSO (73) Na-AnSO (89) Na-PySO (94) PEG600 (25.7) PEG5000 (96.1) PEG20000 (99.6)
[16]	Q_I_	PE MF	Hot-pressing	H_2_O	65	0.75	40 μm	0.086 Lm^−2^ h^−1^ bar^−1^ μm	Ethidium Red (99.9) PEG-600 (99.9) Sucrose (99.9) Glucose (96) Glycerol (53) EG (38) NaCl (95) MgCl_2_ (99.3) CaCl_2_ (99.3)
[35]	Q_I_	PE MF	Hot-pressing	H_2_O	60	0.86	40 μm	0.054 Lm^−2^ h^−1^ bar^−1^ μm	NaCl (94) KCl (92) MgCl_2_ (95) CaCl_2_ (96.9) Sucrose (97.9) Glucose (95) Glycerol (45) Ethylene glycol (38)
[9]	Q_I_	PE MF	Hot-pressing	H_2_O	65	0.75	40 μm	Using Ref. [16]’s LLC membrane. Water filtration performances in between that of commercial RO membranes and NF membranes	
[63]	Q_I_	PES UF	Rod-coating	Glycerol	70	0.96	3 μm	0.054 Lm^−2^ h^−1^ bar^−1^ μm	Sucrose (97) Glucose (87) Glycerol (45) EG (24) NaCl (98) MgCl_2_(99)
[74]	Q_I_	PES UF	Rod-coating	Glycerol	70	≈1	3 μm	Using Ref. [63]’s LLC membrane. Anion exchange in the pores can adjust the flux with little change in rejection performance	
[75]	Q_I_	PES UF	Rod-coating	Glycerol	70	≈1	3 μm	Using Ref. [63]’s LLC membrane. TFC Q_I_ possesses a similar performance as commercial RO and NF membranes in treating hydraulic fracturing flowback water. Controllable DOC recovery can be adjusted by pH	
[76]	Q_I_	PES UF	Rod-coating	Glycerol	70	≈1	3 μm	Using Ref. [63]’s LLC membrane. The 66 h cross-flow filtration of hydraulic fracturing produced water was conducted. Better performance than NF90 in portion of organic compounds, water flux, and fouling resistance	
[68]	H_II_ L_α_	PE MF (recovered)	Hot-pressing	H_2_O	RT (UV-curing) + 70 (thermal-curing)	4 nm (H_II_) 3 nm (L_α_)	10 μm	These LLC membranes possess better permeability and antifouling performances than commercially UF membranes. BSA rejection higher than 95%	
[12]	H_I_	PAN UF	Pressing	H_2_O	RT	1–2 nm	3–30 μm	10 Lm^−2^ h^−1^ bar^−1^ μm	Methylene blue (90) Crystal violet (90) Alcian blue (90) Charged solutes (~350 Da) Neutral solutes (~4 kDa)
[10]	H_I_	PAN UF and PVDF UF	Spin-coating	Glycerol	RT	~1 nm	~200 nm	2 Lm^−2^ h^−1^ bar^−1^ μm	PEG600(>94) Methyl orange (91) Methylene blue (95) CaCl_2_ and MgCl_2_ (>80) LiCl, NaCl, and KCl (>40)
[72]	H_I_	PVDF UF	Spin-coating	Glycerol	RT	0.6–1.5 nm	170–200 nm	10–30 Lm^−2^ h^−1^ bar in water 2–8 Lm^−2^ h^−1^ bar^−1^ μm in methanol	4 H_I_ membranes possess various performances. Only list the maximum performances here. PEG 600 (100%) Acid Fuchsin 585 Da (> 95%) Methyl Orange 327 Da (100%)

**Table 2:** Surface grafting techniques.

Physical Techniques		
Techniques	Principle	Features
Plasma	Interactions between ionized gas with polymer surface atoms, inducing the homolytic bond cleavage, and creating free radicals.	Hydrophobic recovery on a low-surface-energy polymer after simple treatment [148]. The energy required for bond cleavage in the substrate’s material inversely is correlated with the plasma modification efficacy (e.g., C-S is easier than C-C) [149,150]. Hydrophilic modification permanence is correlated with the polymer backbone rigidity (e.g., PES is longer than PE) [149,151]. The grafting efficiency of plasma-induced surface grafting is higher than that of ATRP and thermally induced [152].
UV light	Activating the membrane surface and inducing radical formation by UV irradiation, forming hydrophilic groups with oxygen.	Requiring relatively low mild reaction conditions and low cost equipment [153]. Radical can be directly formed on PES and PSf without a photo-initiator, but a higher degradation degree occurs on these materials [154]. Except for PSf and PES, most of other materials are not photosensitive enough and thus need initiator to form radicals. (e.g., PAN, PP, PE, PVDF and PET) [155]. Grafting can be implemented in both liquid and vapor phases [156].
High-energy irradiation	Activating the membrane surface and inducing radical formation by X-ray, γ-ray and electron source [157,158].	Requirement of radiation source. No requirement of initiator. Grafting occurs within the pore of substrate due to the high penetration [159].
Chemical techniques		
Natively initiating	Grafting small molecules with the left free amine and carboxylic acid groups on polyamide membrane.	This grafting can only be used in polyamide NF and RO membranes.
Surface-initiated atom transfer radical polymerization (SI-ATRP)	Initiator moieties covalently attaches the substrate surface and reacts with the dormant species and induces the ATRP of monomers [160] (grafting from).	Simple experimental setup with easily available initiators. Initiators must be covalently attached on surface first. This method is suitable for growing polymer chains on a variety of material surfaces, including polymers, metals, silicon, metal oxides biologicals species. One can precisely control the grafting density and polymer brushes’ topology and composition [161,162].
Click chemistry	Facilitating the alkyne-azide, thiol-vinyl addition, thiol-yne, etc., reaction to effectively functionalize the membrane surface (grafting to).	Catalyst-free, high-efficiency reaction, which promotes the grafting efficiency of the “grafting to” method [163]. Installation of clickable function groups on the substrate surface is the first concern [164]. Mild reaction temperature (25–70 °C) [165].
Ozone oxidation	Ozone induces the peroxide formation on polymer surface, whose decomposition brings radicals to the surface.	Not suitable for the materials with low surface energy (e.g., PTFE) [166]. Decreasing the membranes’ robustness after long ozone treatment [167].

## Data Availability

Not applicable.
